# Dengue and the Lectin Pathway of the Complement System

**DOI:** 10.3390/v13071219

**Published:** 2021-06-24

**Authors:** Romchat Kraivong, Nuntaya Punyadee, M. Kathryn Liszewski, John P. Atkinson, Panisadee Avirutnan

**Affiliations:** 1 Molecular Biology of Dengue and Flaviviruses Research Team, National Center for Genetic Engineering and Biotechnology, National Science and Technology Development Agency, Bangkok 12120, Thailand; romchat.kra@biotec.or.th; 2 Siriraj Center of Research Excellence in Dengue and Emerging Pathogens, Faculty of Medicine Siriraj Hospital, Mahidol University, Bangkok 10700, Thailand; nuntayap@gmail.com; 3 Division of Dengue Hemorrhagic Fever Research, Faculty of Medicine Siriraj Hospital, Mahidol University, Bangkok 10700, Thailand; 4 Division of Rheumatology, Department of Medicine, Washington University School of Medicine, Saint Louis, MO 63110, USA; kliszews@wustl.edu (M.K.L.); j.p.atkinson@wustl.edu (J.P.A.)

**Keywords:** dengue virus, dengue fever, dengue hemorrhagic fever, dengue shock syndrome, flavivirus, lectin complement pathway, nonstructural protein NS1

## Abstract

Dengue is a mosquito-borne viral disease causing significant health and economic burdens globally. The dengue virus (DENV) comprises four serotypes (DENV1-4). Usually, the primary infection is asymptomatic or causes mild dengue fever (DF), while secondary infections with a different serotype increase the risk of severe dengue disease (dengue hemorrhagic fever, DHF). Complement system activation induces inflammation and tissue injury, contributing to disease pathogenesis. However, in asymptomatic or primary infections, protective immunity largely results from the complement system’s lectin pathway (LP), which is activated through foreign glycan recognition. Differences in N-glycans displayed on the DENV envelope membrane influence the lectin pattern recognition receptor (PRR) binding efficiency. The important PRR, mannan binding lectin (MBL), mediates DENV neutralization through (1) a complement activation-independent mechanism via direct MBL glycan recognition, thereby inhibiting DENV attachment to host target cells, or (2) a complement activation-dependent mechanism following the attachment of complement opsonins C3b and C4b to virion surfaces. The serum concentrations of lectin PRRs and their polymorphisms influence these LP activities. Conversely, to escape the LP attack and enhance the infectivity, DENV utilizes the secreted form of nonstructural protein 1 (sNS1) to counteract the MBL effects, thereby increasing viral survival and dissemination.

## 1. Introduction

Dengue is an insect-borne viral infection transmitted to humans from the bites of infected *Aedes* mosquitoes. The causative agent is dengue virus (DENV), an enveloped positive-sense RNA virus of the *Flaviviridae* family. In contrast to other flaviviruses, it comprises four distinct serotypes (DENV1-4). According to the World Health Organization (WHO), the global incidence of infection has increased dramatically in recent decades [1], and there are an estimated 100–400 million cases per year [2]. Dengue is endemic in the tropical and subtropical regions of the world [3,4]. The majority of infections (>90%) are asymptomatic. However, others present with symptomatic illness ranging from mild dengue fever (DF) to more severe diseases (<5%) known as dengue hemorrhagic fever (DHF) and dengue shock syndrome (DSS) [5]. Typically, symptomatic dengue begins from two to seven days after infection with flu-like symptoms that include fever, headache, myalgias, arthralgias and a maculopapular rash. Hemorrhagic phenomena and leukopenia are common, and thrombocytopenia may also occur (up to 50% in DF and 100% in DHF) [6]. Patients may also exhibit coagulopathy, vascular permeability, hypovolemic shock, bleeding and organ failure, leading to death [4]. While the first or primary infection of DENV is usually asymptomatic or mild, a second infection from a different dengue serotype has an increased risk of severe illness [7,8,9].

Viral virulence and genetic variations lead to different presentations of dengue illness [10]. The introduction of a more virulent Southeast Asian DENV2 to the Americas was responsible for an increased incidence of severe dengue in Cuba in 1981 [8]. As noted above, the temporal sequence of infections, especially with particular serotypes, also correlates with the dengue severity. Epidemiologic studies have shown an association of DHF after a primary infection with DENV1 followed by a secondary infection with DENV2 or DENV3 [7,8,11,12,13].

In addition, multiple host factors determine the disease severity, such as age and ethnicity. For example, in a study of Asian children, the illness in secondary infections presented a greater risk of DHF [14]. Other studies demonstrated that patients with advanced age (>60 years old) are at a high risk to develop severe dengue, partly due to comorbidities or a high incidence of monotypic immune status (previously infected by one of the DENV serotypes). This has been evident in low dengue prevalence areas, where secondary infections with a heterologous DENV serotype increasingly occur in the aged populations [15]. A higher incidence of DHF/DSS has also been observed in patients with AB blood [16], while African ancestry is a protective factor against severe dengue [17], suggesting that host genetics also contributes to a person’s propensity for the development of severe symptoms.

Immune responses to DENV modulate the pathogenesis. High levels of circulating cytokines and chemokines (cytokinemia) in association with massive immune activation (hyperinflammation) are commonly observed in individuals with DHF [7,18,19,20,21]. An increased risk of severe dengue during secondary infection has been partially explained by the antibody-dependent enhancement (ADE) of infection and T-cell original antigenic sin; that is, memory B and T cells activated by the first serotype may have less avidity for epitopes of the new infecting serotype [20,22,23]. ADE occurs when pre-existing antibodies (Ab) from a previous infection bind to viral particles of the current infection with a different DENV serotype. These Abs, instead of effectively inhibiting the infecting virus, enhance the viral entry into Fc-receptor-bearing immune cells such as monocytes and dendritic cells, increasing the total viral replication/burden [23,24,25]. Of note, the ADE of severe dengue in humans has been recently reported [23]. The profound expansion of DENV-specific memory T cells (from the first infection) with a low affinity with the infecting DENV serotype may contribute to delayed viral clearance and the enhanced release of proinflammatory cytokines, leading to more severe manifestations [20,26,27,28]. Polymorphisms in genes related to innate and adaptive (humoral and cellular) immune responses, as well as cytokine and chemokine genes, have been shown to influence the susceptibility to DHF/DSS or severe dengue [29,30,31].

Further, while the complement system plays a protective role in the host by limiting viral replication, overactivation can lead to a more severe disease by exacerbating the inflammatory response (reviewed in [32]). A massive complement activation and a marked reduction in plasma complement proteins were first identified in DHF/DSS patients [33]. High levels of the complement anaphylatoxins (C3a and C5a) and the terminal complement complex (sC5b-9) were present in the plasma of patients with severe dengue during a second infection with a different serotype, suggesting an association between complement activation and dengue severity [34,35,36,37]. These findings suggest that complement overactivation plays a role in DHF/DSS pathogenesis. In addition, soluble immune complexes (IC) formed by circulating DENV and DENV-specific antibodies were detected in the circulation of patients during the acute phase of the disease [36,38]. This complex could be opsonized with complement molecules and rapidly trapped by complement receptors (CR1) in red blood cells (RBCs). The complement fixing IC adheres to the cells until IC-bound RBCs traverse the spleen and liver, where IC is removed from RBC and deposited in these tissues [39]. Although this mechanism is important for viral clearance from the circulation, DENV, in the form of IC, probably takes advantage of this opportunity to infect Fc-receptor-bearing cells in the liver and, therefore, disseminates the infection. This hypothesis, however, needs to be further investigated. Indeed, soluble IC activates complement less efficiently than large immune complexes in which anti-DENV antibodies bind to dengue antigens presenting on DENV-infected cell surfaces [40,41]. The DENV envelope (E) and nonstructural protein 1 (NS1) expressed on infected cell surfaces can be targets for antibody binding and efficiently activate the complement, leading to the deposition of membrane-damaging C5b-9 on the infected cell surface and bystander soluble C5b-9 (sC5b-9) complexes [35,42] and our unpublished data. Complement activation by IC formed on the surface of infected cells, leading to cell lysis, has been suggested to be a key protective mechanism to eliminate infected cells [43,44]. Interestingly, NS1, a major secreted viral protein produced from infected cells, can bind to the surface of uninfected cells via an interaction with glycosaminoglycans [45], which can then form immune complexes with specific antibodies purified from patients’ plasma. This can trigger complement activation, as evident by C3dg and C5b-9 deposition (our unpublished data). Unnecessary complement activation on healthy, uninfected cells caused by NS1-anti-NS1 ICs could lead to inflammatory damage in DHF/DSS patients. In vivo studies of flavivirus-infected mice deficient in complement components, however, support the essential roles of the complement system in protecting these mice from infection [46,47,48,49]. Thus, the complement system is a double-edged sword in its capability to protect from dengue yet, also if overactivated, to enhance disease severity. Hitherto, the roles of the complement have been extensively studied in secondary dengue infections when antibodies are stimulated. On the other hand, the immune mechanisms protecting individuals with the asymptomatic or mild disease, particularly in primary infections, remain uncertain. Many aspects of complement activation and its roles in dengue (protection or pathogenesis) remain to be investigated. In this brief review, we will focus on the role of the lectin pathway of complement activation in DENV.

## 2. Lectin Pathway in Dengue

The three pathways of complement activation are the classical (CP), alternative (AP) and lectin (LP) [50]. The CP is primarily activated by antigen–antibody immune complexes, while the AP amplifies C3b deposition, as well as continuously turning over secondary to hydrolysis of complement component C3. The LP initiates activation through the recognition of glycans “specific” to foreign pathogens or dead cells. Mannan-binding (also called mannose-binding) lectin (MBL) and ficolins (1–3) are the major triggers of the LP. The key effectors of complement activation are anaphylatoxins (C3a and C5a), opsonins (e.g., C4b and C3b) and the membrane attack complex (MAC; C5b-9). The anaphylatoxins bind their receptors to promote potent proinflammatory processes and recruit immune cells to the sites of infection. The opsonic fragments C4b and C3b become covalently tagged onto invading microorganisms or infected cells to induce immune adherence and phagocytosis by immune cells through interactions with complement receptors. The MAC, common to all three pathways, is a terminal assembly complex of complement components (C5b-9) formed on the surface of pathogens and infected cells that causes membrane perturbations, including cell lysis.

**Figure 1:**
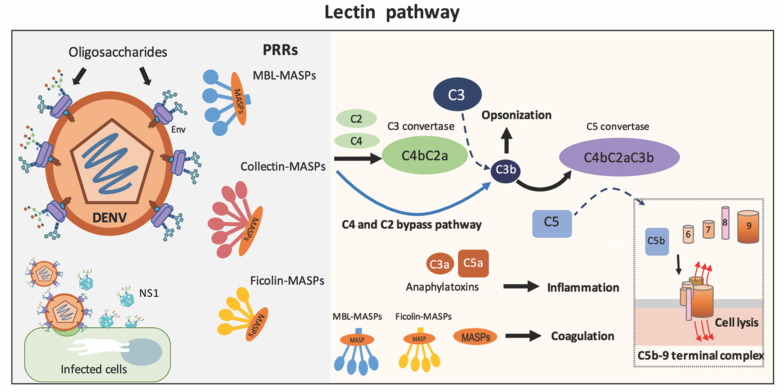
Lectin pathway. Initiation of the lectin pathway (LP) results from the binding of pathogen recognition receptors (PRRs) on microbial carbohydrates, as represented in mature dengue virus. The PRRs (MBL, ficolin, or collectin) assemble with MBL-associated serine proteases (MASP-1 and MASP-2) to activate complement C3 through (1) the actions of C4 and C2 to produce C3 convertase (C4bC2a) or (2) the C4 and C2 bypass pathway. C3b fragments activate further down the cascade to generate C5b-9 complexes on the microbial surface or promote opsonization. C3a and C5a are anaphylatoxins. Additionally, PRR-MASP complexes or MASPs alone are involved in coagulation. Note that the AP can substantially amplify the complement activation from C3b initially generated by the LP.

The LP has been hypothesized to particularly fight against dengue, especially early in primary infections when adaptive immune responses such as specific Abs are lacking and T cells are not yet sensitized [51]. The initiation of this pathway results from the binding of pattern recognition receptors (PRRs) on microbial carbohydrates (e.g., mannose and fucose) or acetylated oligosaccharide residues. Upon binding, the PRRs assemble with MBL-associated serine proteases (MASP-1 and MASP-2) to trigger complement activation. As noted, PRR molecules in the LP include MBL, ficolin-1 (M-ficolin), ficolin-2 (L-ficolin), ficolin-3 (H-ficolin), collectin-10 (CL-10) and collectin-11 (CL-11) [52]. These PRRs and MASPs are predominantly expressed in the liver, a major target organ affected by DENV [53,54]. Of note, among these PRRs, MBL and ficolin-2 have been the most widely studied in infectious diseases such as dengue [55,56,57]. A schematic diagram of the LP is presented in Figure 1.

Animal studies of West Nile Virus (WNV), another flavivirus, have contributed to our knowledge about the protective role of the complement in DENV infection. For example, mice lacking a LP recognition molecule are more vulnerable to WNV infection compared to wild-type mice [46]. Using a panel of naïve sera from mice deficient in a complement component, the neutralization of both WNV and DENV (in the absence of specific antivirus antibodies) was mainly dependent on MBL and MASP-2, partially dependent on factor D and factor B (of the AP) but independent of C1q (the CP) and C5 (MAC formation). These data suggest that, early on in an infection, activation of the CP and the AP might not contribute substantially to direct viral neutralization [47,51]. The serum neutralization of WNV and DENV can also occur via the C4 and C2 bypass pathway; the binding of MBL to the virion’s envelope activates MASPs that directly cleave C3 without the activation of C4 and C2, resulting in the deposition of complement fragments on the pathogen surface (Figure 1) [47,58,59,60]. Although the serum neutralization of both WNV and DENV occurs in a similar manner, certain features of their envelopes differentially affect MBL recognition and, thus, the neutralization efficiency, as further discussed in this review.

### 2.1. Influence of Carbohydrate Structure

Studies on a wide variety of viruses have demonstrated that glycosylation influences the viral virulence. The direct interaction of MBL with flaviviruses, including DENV and WNV, has been demonstrated in vitro [47,51]. MBL recognizes oligosaccharides (glycoproteins) on the virion surface. Flavivirus particles are composed of three structural proteins: envelope (E), membrane (M) and capsid (C) [61]. The E protein serves as the major envelope glycoprotein on the virion surface tasked with virus attachment and fusion onto target cells for productive replication (reviewed in [62]). Virions produced in a mosquito vector versus those in a human host likely have important structural differences in their N-glycans of the E glycoprotein, which could differentially influence the target cell binding and efficiency of infection [63]. The glycans on viruses derived from mosquito cells are primarily high-mannose and/or paucimannose but with terminal mannose residues, while those on virions produced in mammalian cells are mostly complex types, with the exception in DENV, where an additional high-mannose in the second N-glycosylation site is present [62]. Differences in the processing of glycoproteins among host cells can impact the antigenicity and pathogenicity of viruses [29,64,65]. It is worth mentioning that most flavivirus studies have used viruses prepared from infected cells of nonhuman origin, such as C6/36 (mosquito cell line), BHK (baby hamster kidney cell line) and Vero cell (monkey kidney cells line) [45,46,47,51,62], while less studies have used viruses generated from infected human target cells, such as monocytes, monocyte-derived dendritic cells or hepatic cell lines [63,66,67]. This is partly due to a lower yield of infectious virus production. Recently, an in vitro model resembling normal human liver cells that has a high DENV replication efficiency was established [68]. The cells can thus serve as a new alternative cell model to study virus pathogenesis [68].

MBL effectively interacts with the terminal mannoses and thereby preferably recognizes mosquito-derived viruses presenting with simple oligosaccharides on their envelope shells. The direct inhibition of the viruses by MBL, in the absence of a complement activation, is clearly seen in insect cell-derived viruses but less efficiently so on DENV produced by mammalian cells in vitro [51]. This suggests that MBL tends to have greater impact on the neutralization of insect-derived viruses at the initial phase of infection during mosquito inoculation and, to a lesser extent, on human cell-derived viruses produced from sequential rounds of infections. Of note, DENV contains two glycosylation sites at Asn-67 and Asn-153, whereas WNV bears a single N-linked glycosylation site on its E protein at Asn-154 [62]. The effective neutralization of insect cell-derived WNV has been observed in vitro, while little or no binding between MBL and mammalian cell-derived virus results in a less effective viral neutralization [47]. Interestingly, the pretreatment of WNV with deoxymannojirimycin, which prevents the formation of complexes of sugar groups in N-linked glycans, restores the binding and neutralizing ability of MBL to the virus [47]. Furthermore, using genetic engineering to produce a second N-linked glycosylation site at Asn-67 in mammalian cell-derived WNV improved MBL binding and the neutralization of the virus by MBL [47,51]. These findings suggest that MBL binding to flaviviruses is likely modulated by the number and processing of carbohydrates on the N-linked glycans of the E protein.

Unlike WNV, the MBL-mediated neutralization of mammalian cell-derived DENV occurs in the presence of complement activation [51]. The additional high-mannose at Asn-67, unique for DENV, probably influences/facilitates the binding of MBL to DENV and, thus, makes DENV more susceptible to MBL-mediated neutralization when compared with WNV. Strikingly, the enhanced MBL-mediated neutralization of insect cell-derived DENV occurs in the presence of the activation of the LP [51]. A greater number of mannose ligands or the conformational arrangement of the glycans in the mosquito cell-derived virus likely explains this protective phenomenon of the LP. Furthermore, the MBL-dependent neutralization of both mammalian cell-derived and insect cell-derived DENV correlates with the levels of MBL in human serum [51].

**Figure 2:**
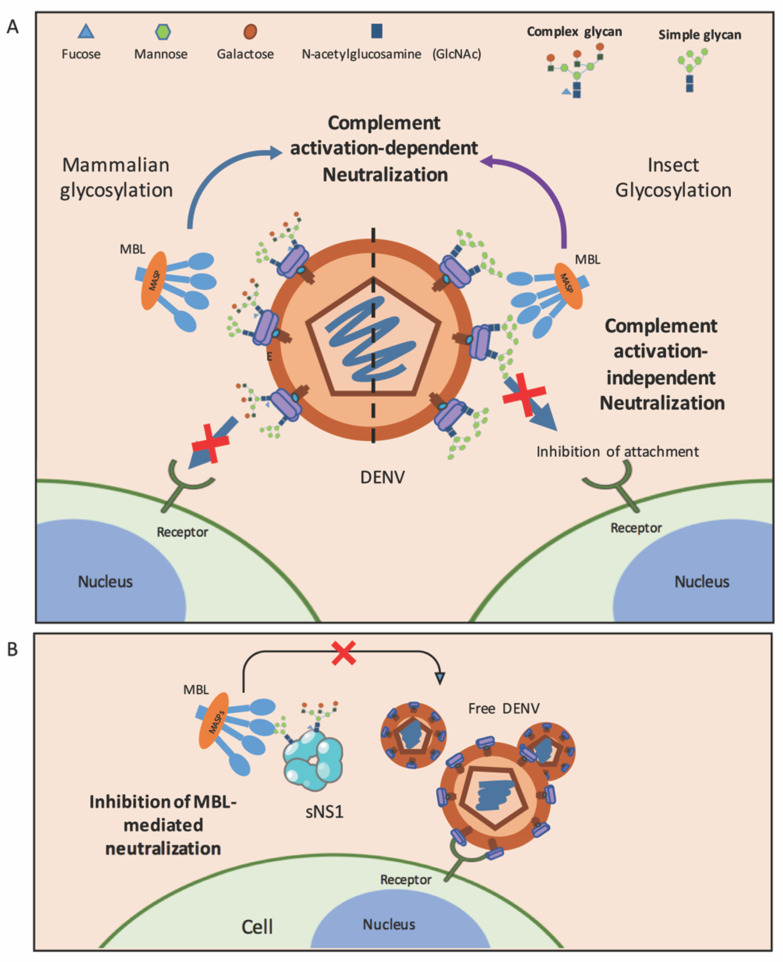
Roles of MBL in dengue. (**A**) MBL molecules inhibit DENV produced from mammalian cells (left) and insect cells (right) via complement activation-dependent or complement activation-independent pathways. Oligosaccharides on the E protein of DENV generated in mammalian cells are complex sugar and high-mannose, while those on the membranes of insect cell-derived viruses are only mannose. (**B**) DENV utilizes sNS1 to counteract MBL in order to escape MBL-mediated neutralization. Free DENV is able to bind to its receptor and infect target cells.

Mechanistically, the MBL neutralization of flaviviruses occurs via two processes: (1) complement activation-independent: the direct interaction of MBL with oligosaccharides on the virion’s envelope shell inhibits viruses from attaching to the host cell membrane—demonstrated for insect and mammalian cell-derived DENV—and (2) complement activation-dependent: the lectin pathway is activated upon MBL binding to DENV and WNV, resulting in the deposition of C3b and C4b on virion surfaces, thereby efficiently enhancing virus neutralization (Table 1 and Figure 2A) [47,51]. Overall, these data suggest that the role of MBL and the lectin activation pathway in human hosts is not only to restrict virus infections after the bite of infected mosquitoes but, also, to control DENV replication.

Of interest, high-mannose residues attached to the E protein on the viral surface bind to dendritic cell-specific ICAM-3 by grabbing nonreceptor integrin (DC-SIGN; CD209) to facilitate the DENV infection of dendritic cells [66,67,70]. The interaction of MBL on DENV may also prevent binding of the virus to DC-SIGN, a major viral entry receptor and the primary target cell for the E protein of DENV, thereby facilitating control of the infection and a diminishing spread in the early phases.

The differential maturation of DENV might also impact MBL-mediated neutralization. The intracellular virus remains in an immature form with the pre-membrane (prM) protein present on the viral envelope. However, shortly before being released into the extracellular milieu, prM is converted into its mature form (M) after cleavage by the host protease “furin”. Consequently, the “pr” peptide is released while the M protein remains attached to the virion surface. Further, the process of furin-mediated prM cleavage induces conformational rearrangements of the surface E and M proteins on its envelope, producing “smooth” outer surfaces of the mature virus [71,72]. The cleavage of prM is, however, ineffective, especially in DENV-infected insect cells. As a result, most extracellular viruses secreted from these cells are “spiky” prM-containing virions, which can be either a partially mature virus or immature virus [71,72,73]. Of note, the prM-retaining virus generated from insect cells could be infectious [24,74]. Thus, MBL binding to prM glycans may also enhance the MBL-mediated neutralization of the insect cell-derived virus. On the other hand, the efficiency of the prM cleavage in DENV-infected mammalian cells appears to differ among cell types. DENV produced from primary human dendritic cells yields much lower levels of the prM protein on its envelope shells than those derived from mammalian cell lines such as Vero (monkey kidney cell line) [24,73,75]. Importantly, DENV prM also contains N-linked glycosylation sites [76], and this could make prM containing immature or partially mature DENV particles more vulnerable to MBL binding and neutralization. The inefficient cleavage of prM on DENV generates a heterogeneous population of mature (virus containing only E and M proteins) and immature particles with different proportions of prM and E [72,77]. Differential numbers of *N*-glycosylation sites on E and prM and the efficiency of prM cleavage by furin (maturation stage) on different viruses that are generated from distinct cell types could influence the degree of complement activation initiated by MBL recognition. However, the impact of these varying outcomes on disease pathogenesis requires further study.

### 2.2. Serum Levels and Polymorphisms of the Lectin PRR Molecules

Low MBL concentrations in DHF patients, particularly with a primary infection, have been demonstrated [78]. This deficiency of MBL in dengue patients who clinically developed DHF after their first encounter with DENV suggests a protective role of MBL in primary DENV infections. Further supporting evidence for this possibility comes from a report that the serum from dengue nonimmune individuals with high MBL concentrations more effectively neutralized the virus than those with low MBL [51]. Additionally, the neutralization of serum MBL by the addition of mannose abrogated the DENV inhibitory capability [51]. Although higher levels of MBL in DHF than those in mild DF cases have been described in another study [34], the collected samples in those experiments were combined from both primary and secondary infections, which may confound the protective effect of MBL, especially relative to severe dengue [34]. In support of this argument, serum from donors who have been previously exposed to the virus effectively neutralized DENV, despite MBL depletion by mannose, indicating a key role of virion-specific Abs, which efficiently trigger the classical pathway of complement activation, resulting in viral neutralization [51].

**Figure 3:**
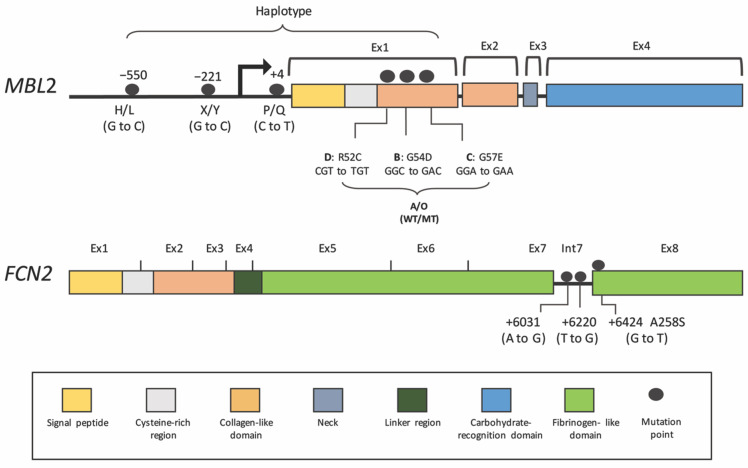
Single Nucleotide Polymorphisms (SNPs) on *MBL2* and *FCN2* genes associated with Dengue. See text and Table 2 and Table 3.

Low-functional MBL in DHF is probably associated with genetic polymorphisms in the *MBL2* gene. Six common single-nucleotide polymorphisms (SNPs) have been studied that are associated with DENV infection (Table 2 and Table 3 and Figure 3) [79]. These variants cause reduced serum levels, as well as changes in MBL function and stability. Located in the *MBL2* promoter are concentration-regulating SNPs (−550:H/L, −221:X/Y and +4:P/Q) of which the −221 locus harboring the X variant has the strongest downregulating effect. The other polymorphisms are in exon 1, which encodes for the structural domains. Mutations in this domain result in amino acid replacements in codon 54 (variant B), codon 57 (variant C) and codon 52 (variant D). The variants B, C and D are often inherited together and are called variant “O”, while a wild-type allele is named allele A. Among these three variants, variant B is the most common in Caucasian (allele frequency = 0.12–0.14) and Asian (allele frequency = 0.12–0.14) populations. The MBL protein produced from these mutants is more easily degraded into lower oligomeric forms and probably has a shorter half-life, leading to reduced function and concentration [80].

Individuals carrying genotype “OO” are considered to be deficient in MBL. In addition, the haplotypes (the combination of SNP alleles on the *MBL2* gene) HYA, LYA and LXA correlate with high, intermediate and low serum MBL levels, respectively [86]. In DENV infections, individuals carrying the “OO” genotype or haplotype for the low MBL level may have a greater risk for the development of DHF, while those with the “AA” genotype tend to have mild dengue disease along with high MBL levels in the blood [51,81,82,83,84]. These findings are different, though, from the observations of Bartolomeu et al. They proposed that individuals carrying the “AA” genotype have a greater chance of developing dengue with thrombocytopenia, and this incidence might increase in adults [85]. Since the genotype “AA” is associated with high concentrations of functional MBL in sera, more MBL–virus complexes could be generated on platelets to promote further complement activation and subsequently induce platelet aggregation and lysis and, thus, thrombocytopenia [52,87]. Of note, dengue virions associated with platelets have been observed both in vitro and in patient blood obtained during the acute febrile phase [88,89]. Intriguingly, complement activation products such as the C3b and C5b-9 complexes have been detected on platelets isolated from dengue patients (Prida Malasit et al., unpublished observation, personal communication).

The interpretation of *MBL2* polymorphisms in DENV infections requires further study. Low frequencies of variant “O” or genotype “OO” in the population limit the power of analysis [86]. In addition, dengue is a complex disease in that analyses must take into account different parameters, such as the classification of dengue infection (1997 WHO versus the 2009 WHO guideline). The type of infection, primary versus secondary, is also very important, as MBL and the lectin complement pathway are likely to be less effective in controlling a virus infection in a secondary immune response in which virus-specific antibodies and memory T cells are rapidly and dramatically mounted in response to a current infection of a person who was exposed to the virus previously. Lastly, the patients’ age (child versus adult) must be taken into consideration. The levels of MBL are normally high in children and decline in adults, and MBL, together with LP activities, also depend on the MBL and MASP2 levels [90,91,92]. In addition, the pathophysiology of DENV infections in children and adults likely differs, as the clinical manifestations and laboratory parameters are distinctly evident between the two age groups [93,94,95,96]. As a result, some of these aforementioned observations may not be definitive, and the data should be interpreted cautiously.

In addition to MBL, other PRRs may have roles in dengue. Recently, the association of ficolin-2 levels, along with *FCN2* polymorphisms in dengue illness, has been examined [55]. The upregulation of ficolin-2 levels during disease progression correlates with DENV infections but not with other febrile diseases of unknown origins (despite lower levels in the blood of severe cases) [55]. Increased ficolin-2 is probably regulated by *FCN2* polymorphisms located in intron 7 (+6031A/G and +6220T/G) and exon 8 (+6424G/T; alanine to serine at codon 258), which might influence the pathogen-binding capacity and concentration (Table 2 and Table 3 and Figure 3) [86,97]. Frequencies of the recessive genotypes in these positions (+6031GG, +6220GG and +6424TT) are high in dengue patients as compared to the controls and are much higher in severe cases [55]. This evidence points to the potential role of ficolin-2 in response to dengue infection, but the mechanisms of how this PRR of the LP behaves towards dengue virus need to be examined in more detail.

### 2.3. Dengue Nonstructural Protein NS1, the Antagonistic Molecule to the Lectin Pathway

Although the LP is an effective mechanism to limit infection, DENV has developed strategies to protect itself from a lectin-mediated attack (Table 1 and Figure 2B). DENV produces a nonstructural protein (NS1) to assist in intracellular viral replication [98,99], enhance dengue infection [100,101,102] and modulate the endothelial hyperpermeability, potentially to disseminate infections [103,104,105,106], which could further contribute to dengue pathogenesis [99,107]. NS1 is a glycoprotein that can be detected as intracellular, surface membrane-associated and secreted forms [45,99]. Interestingly, the secreted NS1 (sNS1) performs immune evasion functions, one of which is to antagonize complement molecules, including MBL [69,108]. Viruses utilize this major secreted molecule to counteract MBL, thereby inhibiting MBL-mediated neutralization (independent of the complement activation) [69]. More viral particles are therefore free to bind to human cells and, thus, increase the viral infectivity. Of note, sNS1 derived from both insect and mammalian cells is capable of binding to MBL [69]. The interaction of MBL with DENV sNS1 might be attributed to the *N*-linked glycosylation sites at Asn-130 and Asn-207 [109,110]. Secreted NS1 from DENV-infected insect cells contain high-mannose glycans, while those propagated in mammalian cells display high-mannose residues at Asn-207 and complex type *N*-linked glycans at Asn-130 [110]. These results imply that sNS1 restricts the complement activities during a natural infection after sNS1 is secreted from mosquito saliva, as well as during subsequent rounds of infection in humans. Of note, this lectin-specific antagonism of sNS1 of DENV, from both insect and mammalian cells, to evade the innate immune system has not yet been reported in other members of the *Flaviviridae* family. More studies using sNS1 prepared from human cells, together with sNS1 derived from the sera of dengue patients, are still needed to confirm the complement evasion function of sNS1 in humans.

The antagonistic functions of sNS1 are not limited to MBL inhibitory activities. NS1 of DENV, WNV and yellow fever virus also bind to the C1s, C4 and C4b-binding proteins, promoting C4 degradation and further attenuating both the LP and CP complement pathways [51,111]. Furthermore, the inhibition of terminal complement complexes by DENV NS1 through interactions with complement regulatory proteins has been described [112,113]. NS1 is thus a protein that is capable of helping DENV escape from an immune attack, thereby increasing the chances of viral survival and dissemination.

## 3. Concluding Remarks and Future Perspectives

In this review, dengue pathogenesis mediated by the LP was emphasized in order to highlight the importance of this pathway. The LP may play a role primarily in protecting hosts from natural DENV infections after mosquito bites, leading to asymptomatic infections. The neutralization of DENV by the LP is both complement activation-dependent and -independent. An underlying mechanism of lectin-mediated neutralization is the prevention of virus attachment to its target cells. However, DENV has developed strategies to escape from lectin recognition. The pattern and type of *N*-linked glycans, as well as differential structures of DENV, can be deceptive for the lectin PRRs. Remarkably, DENV utilizes sNS1 to counteract MBL, thereby enhancing the virus infectivity. Polymorphisms leading to reduced serum levels of lectin MBL and ficolin-2 have been correlated with dengue severity.

The current knowledge about DENV infection and the LP provides only a glimpse of the possible pathologic and defensive mechanisms. Insights into the regulation of the LP in dengue pathogenesis need to be further elaborated. Additional prospective clinical studies, as well as the development of suitable animal models for DENV are necessary for investigating the precise roles of MBL deficiency or allelic variations of MBL in DENV infections. Improved understanding should eventually lead to improved antiviral therapy and vaccine approaches.

## Figures and Tables

**Table 1:** Comparison of MBL-mediated neutralization between DENV and WNV.

Mechanisms	DENV	WNV
N-linked glycosylation site on E protein	Two glycosylation sites at Asn-67 and Asn-153 [62]	One glycosylation site at Asn-154 [62]
MBL-mediated neutralization dependent of complement activation	Neutralization of insect and mammalian cell-derived virus [47,51]	Neutralization of insect cell-derived virus by blocking viral fusion [47]
MBL-mediated neutralization independent of complement activation	Neutralization of insect and mammalian cell-derived virus by inhibition of viral attachment to target cells [51]	-
Mechanism of immune evasion to MBL-mediated neutralization	Both insect cell and mammalian–derived sNS1 bind to MBL, inhibit MBL-mediated neutralization [69]	-

**Table 2:** Single-nucleotide polymorphisms in the *MBL2* and *FCN2* genes associated with dengue disease.

Protein	Gene	dbSNP	Nucleotide Location	Major Allele	Minor Allele	Region	Amino Acid Substitution	References
MBL	*MBL2*	rs11003125 (H/L)	−550	G	C	Promoter	-	[51,81,82,83,84]
		rs7096206 (X/Y)	−221	C	G	Promoter	-	[51,81,82,83,84]
		rs7095891 (P/Q)	+4	C	T	5’ UTR	-	[51,81,82,83,84]
		rs5030737 (Variant D)	+223	C	T	Exon1	R52C	[51,81,82,83,84]
		rs1800450 (Variant B)	+230	G	A	Exon1	G54D	[51,81,82,83,84]
		rs1800451 (Variant O)	+239	G	A	Exon1	G57E	[51,81,82,83,84]
Ficolin−2	*FCN2*	rs11103563	+6031	A	G	Intron 7	-	[55]
		rs7872508	+6220	T	G	Intron 7	-	[55]
		rs7851696	+6424	G	T	Exon8	A258S	[55]

**Table 3:** Allele, genotype and haplotype of the polymorphisms in *MBL2* and *FCN2* associated with dengue disease.

Gene	Polymorphisms	Serum Level	Association to Dengue Disease	References
*MBL2*	Exon 1 Allele			
	O	Low	A greater risk to develop DHF	[81]
	Exon 1 Genotype			
	AA	High	Mild dengue disease; a greater chance to develop dengue with thrombocytopenia	[85]
	OO	Low	A greater risk to develop DHF	
	Diplotype			
	XA/XO, YA/XO	Not available	Association with dengue disease	[84]
	Haplotype			
	LXPB, HXPA, XO	Not available	Association with dengue disease	[84]
	LXA/HYO, LXA/LYO, HYO/LYO	Low	A greater risk to develop DHF	[81]
*FCN2*	Intron 7 and Exon 8 (+6031A/G, +6220 T/G, +6424 G/T)			
	Recessive genotype (6031GG, 6220GG, 6424TT)	High	Susceptibility to dengue disease	[55]

## Data Availability

Not applicable.
